# Improved Glycemic Control through Robot-Assisted Remote Interview for Outpatients with Type 2 Diabetes: A Pilot Study

**DOI:** 10.3390/medicina60020329

**Published:** 2024-02-15

**Authors:** Kunimasa Yagi, Michiko Inagaki, Yuya Asada, Mako Komatsu, Fuka Ogawa, Tomomi Horiguchi, Naoto Yamaaki, Mikifumi Shikida, Hideki Origasa, Shuichi Nishio

**Affiliations:** 1 Department of Internal Medicine, Kanazawa Medical University Hospital, Ishikawa 920-0293, Japan; 2 First Department of Internal Medicine, Toyama University Hospital, Toyama 930-0152, Japan; 3 Faculty of Health Sciences, Institute of Medical, Pharmaceutical and Health Sciences, Kanazawa University, Ishikawa 920-1192, Japan; ja9xbh@yahoo.co.jp (M.I.); y-asada@staff.kanazawa-u.ac.jp (Y.A.); horiguchi@mhs.mp.kanazawa-u.ac.jp (T.H.); 4 School of Informatics, Kochi University of Technology, Kochi 780-8515, Japan; komatsu.21151@gmail.com (M.K.); 240301e@ugs.kochi-tech.ac.jp (F.O.); shikida.mikifumi@kochi-tech.ac.jp (M.S.); 5 Isobe Clinic, Ishikawa 290-0511, Japan; isobe_dm_clinic@yahoo.co.jp; 6 Data Science and AI Innovation Research Promotion Center, Institute of Statistical Mathematics, Shiga University, Shiga 525-0034, Japan; origasahideki@gmail.com; 7 Institute for Open and Transdisciplinary Research Initiatives, Osaka University, Osaka 565-0871, Japan; nishio@botransfer.org

**Keywords:** type 2 diabetes, robot, RoBoHoN, certified diabetes care and education specialists, diabetes self-management, information and communication technology

## Abstract

*Background and Objectives*: Our research group developed a robot-assisted diabetes self-management monitoring system to support Certified Diabetes Care and Education Specialists (CDCESs) in tracking the health status of patients with type 2 diabetes (T2D). This study aimed to evaluate the impact of this system on glycemic control and to identify suitable candidates for its use. *Materials and Methods*: After obtaining written informed consent from all participants with T2D, the CDCESs conducted remote interviews with the patients using RoBoHoN. All participants completed a questionnaire immediately after the experiment. HbA1c was assessed at the time of the interview and two months later, and glycemic control status was categorized as either “Adequate” or “Inadequate” based on the target HbA1c levels outlined in the guidelines for adult and elderly patients with type 2 diabetes by the Japan Diabetes Society. Patients who changed their medication regimens within the two months following the interview were excluded from the study. *Results*: The clinical characteristics of the 28 eligible patients were as follows: 67.9 ± 14.8 years old, 23 men (69%), body mass index (24.7 ± 4.9 kg/m^2^), and HbA1c levels 7.16 ± 1.11% at interview and two months later. Glycemic control status (GCS) was Adequate (A) to Inadequate (I): 1 case; I to A: 7 cases; A to A good: 14 cases; I to I: 6 cases (*p*-value = 0.02862 by Chi-square test). Multiple regression analyses showed that Q1 (Did RoBoHoN speak clearly?) and Q7 (Was RoBoHoN’s response natural?) significantly contributed to GCS, indicating that the naturalness of the responses did not impair the robot-assisted interviews. The results suggest that to improve the system in the future, it is more beneficial to focus on the content of the conversation rather than pursuing superficial naturalness in the responses. *Conclusions*: This study demonstrated the efficacy of a robot-assisted diabetes management system that can contribute to improved glycemic control.

## 1. Introduction

The management of type 2 diabetes (T2D) involves multifaceted challenges, particularly in self-management, which includes diet, exercise, and adherence to medication [1,2]. Certified Diabetes Care and Education Specialists (CDCESs) play a pivotal role in this sense [3], employing a comprehensive approach to assess and enhance patients’ knowledge, behaviors, and support systems [4]. The effectiveness of CDCES in facilitating behavioral change, enhancing glycemic control, and preventing diabetes-related complications is well established, reflecting their crucial role in improving the quality of life of individuals with T2DM [5].

However, the increasing global prevalence of diabetes, with an estimated 463 million adults living with the condition in 2019 and projections suggesting a rise to 700 million by 2045 [6], has led to a considerable gap in healthcare provision. This burgeoning epidemic is outstripping the availability of skilled CDCES, especially in Japan’s super-aged society [7]. The uneven distribution of CDCES, predominantly concentrated in urban medical centers and university hospitals rather than in rural private clinics, exacerbates this shortfall. Additionally, the COVID-19 pandemic has exacerbated this situation by limiting face-to-face interactions in medical settings, thereby hindering traditional methods of diabetes education and care.

In response to these challenges, significant global investment has been directed toward developing information and communication technology (ICT) systems to support diabetes management. Our research group acknowledges that although these technological advancements are promising, the absence of human empathy and interaction in computer-mediated education frequently results in less-than-ideal patient outcomes. To address this, we developed a novel robot-assisted diabetes self-management monitoring system [8,9,10]. This system, designed to support CDSESs in various tasks such as information gathering and patient education, includes robots that can mimic human expressions. This feature allows for a more engaging and personable approach to providing diabetes nutritional guidance.

Introducing such expressive robots bridges the gap between technological innovation and empathetic patient care. By doing so, we revolutionize patient engagement and transform the landscape of diabetes education. This study evaluated the impact of our system on glycemic management and identified suitable candidates for this technology. Our approach addresses the shortfall in CDSES availability. It challenges the belief that significant investments in medical ICT do not necessarily result in practical medical improvements, potentially leading to considerable decreases in future healthcare expenses and improving the well-being of individuals with T2D.

## 2. Materials and Methods

### 2.1. Study Design and Ethical Issues

This is a retrospective cross-sectional observational study of a hospital-based cohort. All procedures followed the ethical standards of the responsible committee on human experimentation and the Helsinki Declaration of 1964, as well as its later amendments. The study protocol was approved by the Ethics Committee of Toyama University Hospital (IRB# R2021083). We obtained written informed consent from all participants, informing them that they could opt out at any time.

### 2.2. Study Population

This study analyzed data from patients with T2D to assess their glycemic control status (GCS) at the Kitano Internal Medicine Clinic (Kanazawa, Japan), between November 2021 and October 2022. All patients in the study cohort regularly attended the clinic monthly or bimonthly. Even for those attending monthly, the initial HbA1c measurement was confirmed two months after the robotic interview.

The inclusion criteria included patients with T2D who had their HbA1c levels evaluated at baseline and again two months later. The exclusion criteria were as follows: (i) type 1 diabetes, (ii) secondary diabetes, (iii) refractory malignant diseases, (iv) dependency on hemodialysis, (v) renal dysfunction with serum creatine levels over 2.5 mg/dL, (vi) symptomatic coronary artery disease or percutaneous coronary intervention within the past year, (vii) severe hepatic dysfunction (Child–Pugh score ≥10), and (viii) patients who had changed their prescriptions within the two months prior to the interview.

### 2.3. Definitions

T2D diagnoses were established based on the diagnostic criteria outlined by the American Diabetes Association and The Japan Diabetes Society (JDS), involving the presence of HbA1c levels ≥6.5% (National Glycohemoglobin Standardization Program), fasting blood glucose concentrations ≥126 mg/dL (7.0 mmol/L), random blood glucose concentrations ≥200 mg/dL, or the current use of medications for diabetes [11,12].

Diabetic nephropathy is defined by urinary albumin excretion ≥30 mg/g creatinine or an estimated glomerular filtration rate < 60 mL/min/1.73 m^2^ [11,12].

GCS was classified as Adequate (A) or Inadequate (I) based on HbA1c levels according to the JDS treatment goal guidelines for adult and elderly patients with T2D, considering age and activities of daily living for the elderly [11].

### 2.4. Robot-Assisted Interview

**Figure 1:**
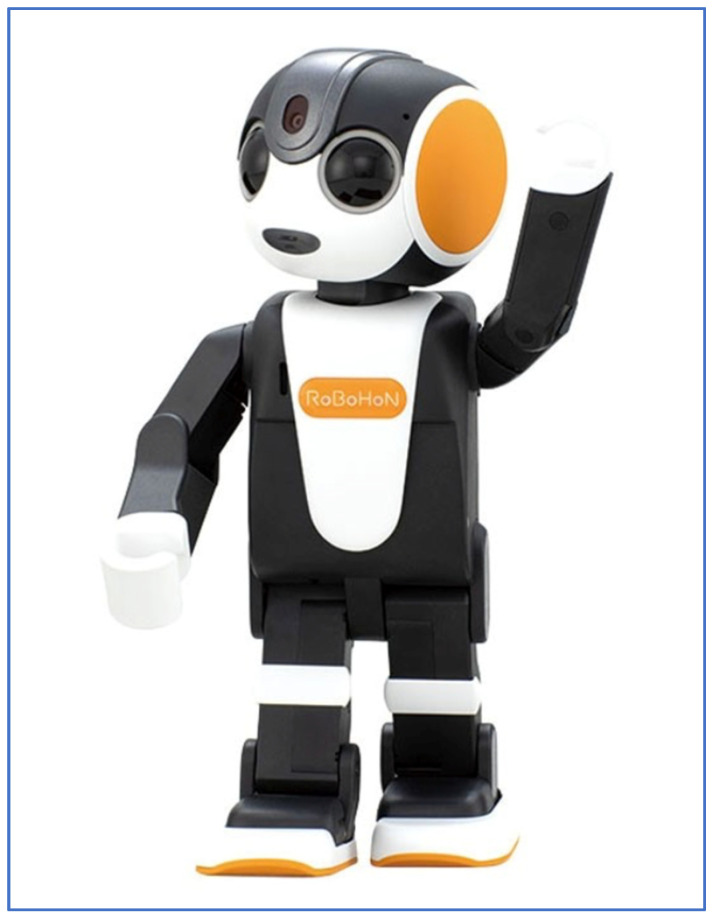
RoBoHoN image from https://cocorostore.sharp.co.jp/robohon/ (accessed on 1 January 2024). (In Japanese).

The development of a robot-based remote medical interview system was motivated by the unique capabilities of robotic technology for providing diabetes care guidance. Detailed information on this innovative system can be found in a separate publication [13]. In brief, this system involves the remote operation of a robot by a CDCES to gather relevant information regarding the patient’s diabetes self-management, including knowledge related to diet and exercise, behavioral patterns, and the support environment. All responses provided by the robot are preprogrammed in its control system prior to the interaction, allowing the CDCES to communicate precise questions and acknowledgments to patients with diabetes at the touch of a button. Patient responses are recorded using a camera and microphone embedded in the RoBoHoN (Sharp Corporation, Japan) [14] (Figure 1), enabling the CDCES to retrospectively analyze the conversation. This innovative approach improves the efficiency and effectiveness of remote diabetes management by incorporating robotic technology into the healthcare delivery process.

### 2.5. Evaluation of Robot-Assisted Interviews by Certified Diabetes Care and Education Specialists

Two CDCESs, Y.A. and M.I., actively participated in this research endeavor. Their combined experience as CDCESs spans 12 and 38 years, respectively, contributing valuable insights to the system’s development, as previously detailed. Beyond their involvement in the system’s development, these CDCES independently assessed the self-care behaviors in patients with T2DM.

### 2.6. Assessment of Robot-Assisted Interviews with Participating Individuals with Diabetes

The effectiveness of the robot-assisted interviews was evaluated by administering questionnaires immediately after the interviews, with the relevant questions categorized under Quality of Care (QC) (Table 1). These questionnaires aimed to assess the perceived usefulness and efficacy of the interview method, offering important perspectives on the patient experience and the overall influence of the robot-assisted approach on healthcare interactions. The specific QC items on the questionnaire addressed various aspects of the interview, highlighting its thoroughness, patient engagement, and potential areas for improvement.

### 2.7. Statistical Analysis

The sample size was calculated using Lehr’s formula, aiming for a power of 80% and a significance level (α) of 0.05, resulting in a required sample size of 32 patients. Continuous variables were expressed as mean ± SD and median, and categorical variables were expressed as numbers and percentages. Continuous variables were compared using an independent samples *t*-test. A comparison of the categorical variables between the groups was performed using a chi-square test and Kruskal-Wallis test. Multivariate liner regression analysis was conducted to evaluate the predictive effect of robot usage on questionnaire outcomes, adjusting for other potential confounders. Statistical analyses were performed using JMP ver. 16.1.2000. (SAS Institute Inc., Cary, NC, USA), R 4.3.0 GUI 1.79, and R studio ver. 2023.06.0 + 421 (Boston, MA, USA) on a Macintosh computer.

## 3. Results

We obtained written informed consent from the 33 patients with diabetes. Three participants were unable to complete the follow-up data collection at the two-month mark. Two participants were excluded due to changes in their oral medication regimen. Consequently, the analysis incorporated data from 28 participants, and their clinical characteristics are summarized in Table 1. In summary, the cohort had a mean age of 69.5 ± 12.7 years, included 20 males (71%), and had a mean body mass index (BMI) of 24.9 ± 4.9 (Table 2). Pharmacological treatments for diabetes were as follows: three cases with insulin injections, seven cases with GLP-1 analog injections, seven cases with biguanides, ten with insulin secretagogue, eighteen with SGLT2 inhibitors, sixteen with DPP4 inhibitors, and three with thiazolidine, and three with imeglimin. Throughout the study, all patients demonstrated sufficient compliance with the interview process.

All patients demonstrated sufficient acceptance of the system during the completion of the RoBoHoN-assisted interview. At the time of the interview, the mean HbA1c level was 7.06 ± 0.69%; two months later, it was 6.86 ± 0.62%. Notably, after two months, participants who underwent RoBoHoN interviews exhibited a 0.19 ± 0.29 percentage point reduction in HbA1c. The distribution of changes in GCS was as follows: A to I: 1 case; I to A: 7 cases; A to A: X cases; I to I: X cases (ChiSq *p* < 0.0001) (Table 3). Fisher’s exact test indicated a significant difference in status, with seven instances improving from I to A and one case deteriorating from A to I (*p* = 0.02862).

**Figure 2:**
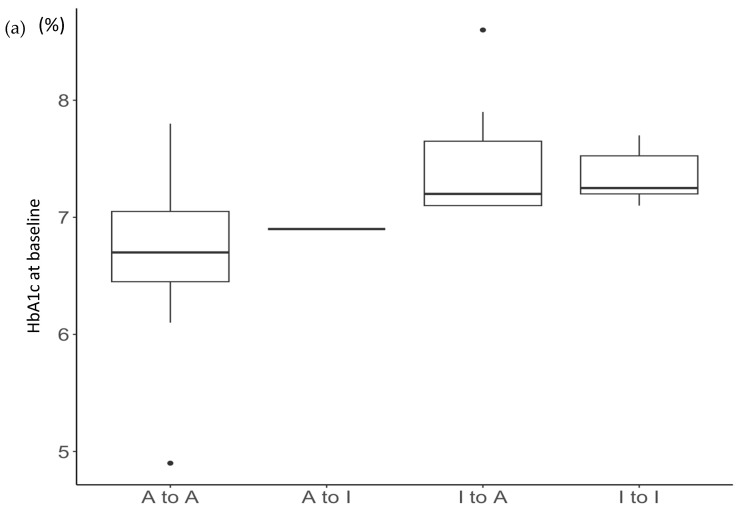

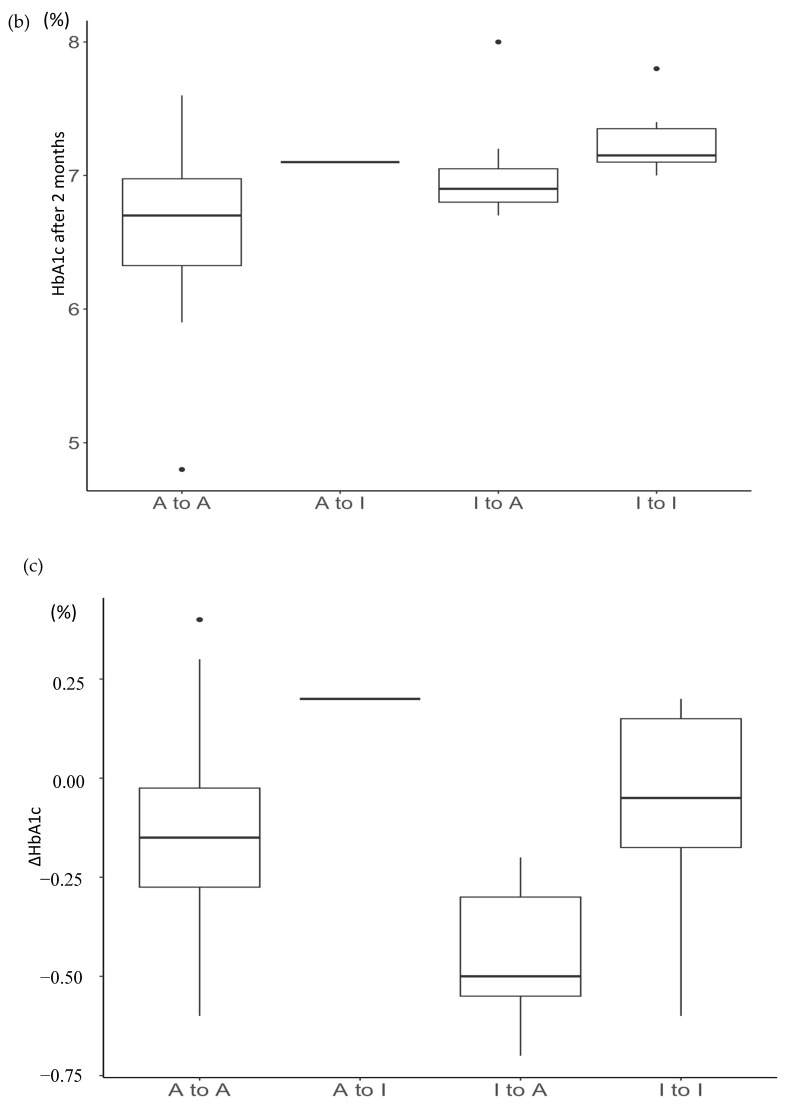

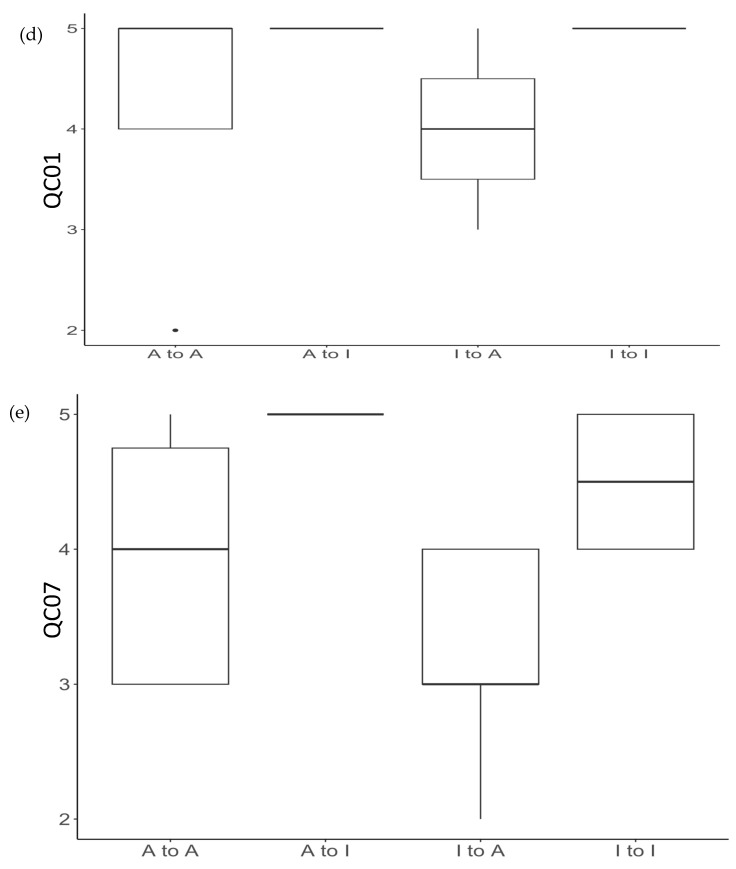

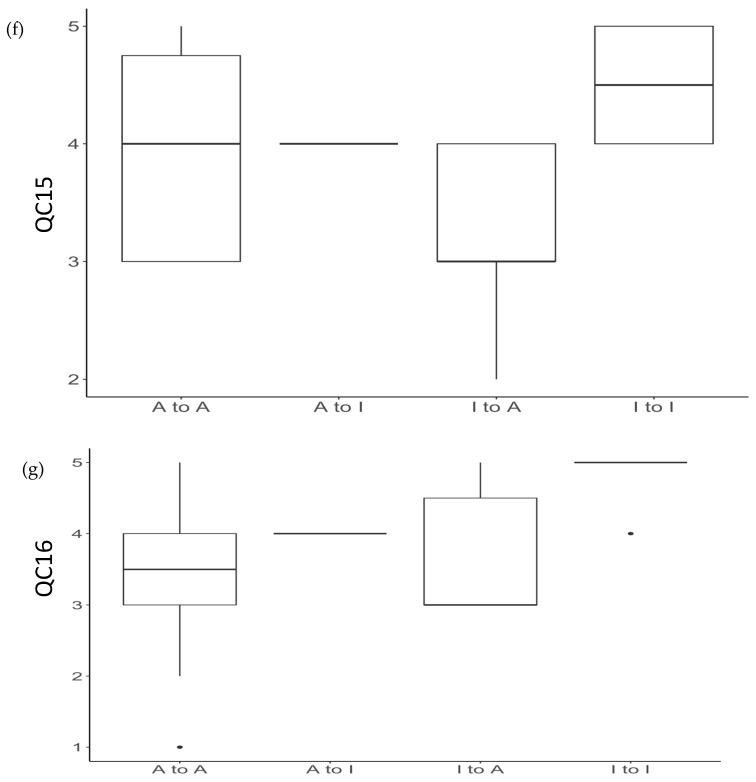
Relationship between the change in glycemic control and the following parameters: (**a**) baseline HbA1c; (**b**) HbA1c two months post-intervention with the RoBoHoN-mediated interview; (**c**) change in HbA1c from baseline to two months post-intervention; (**d**) QC01; (**e**) QC07; (**f**) QC15; (**g**) QC16.

Because the I to A (Adequate to Inadequate) group consisted of only one case, it was combined with the I to I (Inadequate to Inadequate) group, which had six cases. Then, among the three groups of this A to I + I to I, I to A (Inadequate to Inadequate), and A to A (Adequate to Adequate), a Kruskal–Wallis test revealed significant factors such as HbA1c (0M), HbA1c (2M), ∆HbA1c, QC01 (Did RoBoHoN speak clearly?), QC07 (Was RoBoHoN’s response natural?), QC15 (Did you feel more pressure when tested during a conversation with a robot phone than when communicating with a healthcare provider?), and QC16 (Did you feel uncomfortable discussing personal matters because of RoBoHoN’s childish way of speaking?) (Table 4). Figure 2 shows the distribution of each factor across the four groups of HbA1c at baseline and two months post-intervention and its changes, QC01, QC07, QC15, and QC16.

By further exploring potential contributing factors, we have decided to retain QC01 and QC07 as significant predictors in the model (Table 5).

## 4. Discussion

Despite the absence of any deliberate interventions, instances where the GCS category improved outnumbered those where it deteriorated. When classifying groups based on the presence or absence of improvement, significant differences in response rates were observed for Question 1 (Did RoBoHoN speak clearly?) and Question 7 (Was RoBoHoN’s response natural?). Paradoxically, participants who experienced difficulties in understanding the robot’s speech and perceived its reactions as unnatural demonstrated improvement in the GCS category.

The participants in this study, with a mean age of 69.5 years, mean BMI of 24.9, and mean HbA1c of 7.06%, closely resemble the demographic characteristics reported in the 2022 JDS JDDM study (mean age of approximately 67.71 years, mean BMI of 24.74, mean HbA1c 7.14%) [15]. This comparison aligns our study population with the broader context of diabetes management in the Japanese population.

The JDS has recently recommended specific glycemic control targets for the elderly, considering factors such as age, functional independence, and medication regimens [11,16]. This approach recognizes that the target HbA1c levels can differ depending on individual circumstances. According to these guidelines, both avoiding hypoglycemia and reducing HbA1c levels were prioritized, with target ranges set for HbA1c, indicating that drastic reductions in HbA1c levels are undesirable. In the cases presented here, none fell below the minimum threshold.

In this study, the robots were strictly limited to data collection without any form of intervention, such as providing medical advice or educational instruction. Although standard practices during routine medical consultations by healthcare professionals often include similar questions, these interactions can become monotonous and may not adequately increase the awareness of individuals managing diabetes. It is presumed that individuals were given the opportunity to reflect on factors such as medication adherence, and eating habits between meals through interactions with robotic inquiries.

Previous research assessing interactions between robots and the elderly has indirectly identified positive impacts [17,18,19,20]. Even in interactions with elderly individuals, there was no proactive intervention; instead, the approach remained reactive. In this context, structured inquiries by the robot may have triggered increased awareness among patients, offering a new perspective on the potential benefits of robotic engagement in healthcare environments.

An intriguing aspect of our findings is the responses to questionnaire items QC01 and QC07. Notably, individuals who initially found it challenging to comprehend the robot’s speech showed improvement, as did those who initially perceived the robot’s responses as unnatural. Equally noteworthy is the lack of significant differences among the three groups regarding retrospective questions (Q18–20) pertaining to reflection. Contrary to conventional expectations, the observed glycemic improvements were not associated with the reflection effects in this study.

Considering QC15 and QC16 together, it is reasonable to hypothesize that individuals who engaged in the interaction with genuine intent to “listen as they would to a human” have experienced improvement. Interestingly, patients who interacted with the robot as if it were a human, despite potential discomfort, seemed to manage the experience smoothly. These findings indicate that instead of prioritizing the superficial naturalness of responses, future system enhancements may benefit more from refining the content and direction of the conversation.

This study has several limitations due to its single-center design and private clinic setting, which inherently limit the generalizability of the findings. However, this study concentrates on examining the potential benefits and practical applications of the robot, acknowledging that although there are limitations, the current dataset offers valuable insights into its effectiveness in a specific healthcare environment.

## 5. Conclusions

This study indicates that robot-assisted remote diabetes education enhances the support provided by CDCESs to outpatients with T2D, and this approach is applicable to the elderly living in rural regions of Japan. However, it should be tested more extensively.

## Figures and Tables

**Table 1:** Questionnaires evaluating diabetes self-care status, categorized under Quality of Care.

Category 1: Functional quality of RoBoHoN-mediated interview	
QC-01	Did RoBoHoN speak clearly?
QC-02	Did RoBoHoN speak at an appropriate speed to be understood?
QC-03	Do you feel satisfied that you have told RoBoHoN what you wanted to say?
QC-04	Did RoBoHoN ask questions in timely and in natural?
QC-05	Were RoBoHoN’s questions difficult to understand?
QC-06	Did RoBoHoN reply in a natural time?
QC-07	Was RoBoHoN’s response natural?
Category 2: Impression of RoBoHoN	
QC-08	Was RoBoHoN cute?
QC-09	Did you feel afraid of RoBoHoN (scary, cold, etc.)?
QC-10	Did you feel attached to RoBoHoN while talking with him?
QC-11	Did you feel familiar with the way RoBoHoN speaks?
QC-12	Did you want to talk more with RoBoHoN?
Category 3: Advantages of RoBoHoN-mediated interviews over usual CDCESs’	
QC-13	Did you feel more comfortable talking to RoBoHoN than to a medical professional?
QC-14	Was it easier to talk about difficult things with RoBoHoN than with a medical professional?
QC-15	Did you feel more pressure of tested when talking to a RoBoHoN than when communicating with a medical professional?
QC-16	Did you feel uncomfortable discussing personal matters because of RoBoHoN’s childish way of speaking?
QC-17	Did you feel that coming to the clinic was more fun?
Category 4: RoBoHoN’s effect on reflection on diabetes self-care	
QC-18	Did your conversation with RoBoHoN make you reflect on your own knowledge about diabetes?
QC-19	Did your conversation with RoBoHoN give you an opportunity to reflect on your own diet and eating habits regarding diabetes?
QC-20	Did your conversation with RoBoHoN give you an opportunity to reflect on your own activity level and exercise habits regarding diabetes?
QC-21	Did your conversation with RoBoHoN cause you to reflect on medications (oral medications and insulin) in relation to diabetes?
QC-22	Did your conversation with RoBoHoN cause you to reflect over your glycemic management?

CDCES, Certified Diabetes Care and Education Specialists; QC, questionnaires categorized under Quality of Care. Each questionnaire should be answered in five degrees: strongly disagree, disagree, neutral, agree somewhat, and strongly agree.

**Table 2:** Baseline characteristics of the participants.

Characteristics	Variables
Number of subjects	28
Age, years old	69.5 ± 12.7, 70.5 [63.75, 79.25]
Gender (Male/Female)	20/8
HbA1c (0M), %	7.06 ± 0.69, 7.1 [6.75, 7.45]
HbA1c (2M), %	6.86 ± 0.62, 6.9 [6.675, 7.125]
ΔHbA1c, %	−0.19 ± 0.29, −0.2 [−0.4, 0]
Glycemic control (0M) Adequate/Inadequate	15/13
Glycemic control (2M) Adequate/Inadequate	21/7
BMI, Kg/m^2^	24.9 ± 4.9, 24.2 [22.25, 27.4]
Number of diabetes medications	2.1 ± 1.1, 2 [1,3]
Subjects with diabetic complications	5
Treated with insulin injections	3 (11%)
Treated with GLP-1 analog injections	7 (25%)
Treated with biguanide	7 (25%)
Treated with sulfonylureas and glinides	10 (36%)
Treated with SGLT2 inhibitors	18 (64%)
Treated with DPP-4 inhibitors	16 (57%)
Treated with thiazolidine	3 (11%)
Treated with imeglimin	3 (11%)
Grade of diabetes care behavior (6–18)	9.50 [8.00, 12.00]
Understanding of diabetes therapy (3–9)	4.00 [3.75, 5.00]
Acceptance of diabetes (3–6)	4.00 [4.00, 5.00]
Effectiveness of Robot interview (2–4)	4.00 [4.00, 4.00]
QC-01 (1–5)	5.00 [4.00, 5.00]
QC-02 (1–5)	5.00 [4.00, 5.00]
QC-03 (1–5)	4.00 [4.00, 5.00]
QC-04 (1–5)	4.00 [3.75, 5.00]
QC-05 (1–5)	3.00 [3.00, 4.00]
QC-06 (1–5)	4.00 [3.00, 5.00]
QC-07 (1–5)	4.00 [3.00, 5.00]
QC-08 (1–5)	4.00 [3.75, 5.00]
QC-09 (1–5)	5.00 [4.00, 5.00]
QC-10 (1–5)	3.00 [3.00, 4.00]
QC-11 (1–5)	4.00 [3.00, 5.00]
QC-12 (1–5)	3.00 [3.00, 4.00]
QC-13 (1–5)	3.00 [3.00, 4.00]
QC-14 (1–5)	3.00 [3.00, 4.00]
QC-15 (1–5)	4.00 [3.00, 4.25]
QC-16 (1–5)	4.00 [3.00, 5.00]
QC-17 (1–5)	3.00 [2.75, 3.00]
QC-18 (1–5)	4.00 [3.00, 4.00]
QC-19 (1–5)	4.00 [3.00, 5.00]
QC-20 (1–5)	4.00 [3.00, 4.25]
QC-21 (1–5)	3.00 [3.00, 4.00]
QC-22 (1–5)	4.00 [3.00, 5.00]

BMI, body mass index; GLP-1, glucagon-like peptide 1; M, months; QC, Questionnaires categorized under Quality of Care. Clinical features as age, HbA1c, BMI, and the number of diabetes medications were expressed in mean ± S.D., and median. The scores of diabetes self-care status, and QC01 to QC-22 were expressed as median [interquartile range].

**Table 3:** Change in glycemic management status two months after RoBoHoN-mediated interview.

	Adequate GMS after 2 Months	Inadequate GMS after 2 Months
Adequate GMS at baseline	14	1
Inadequate GMS at baseline	7	6

Odds ratio 10.9 (*p*-value = 0.029 by Fisher exact test). GMS, glycemic management status.

**Table 4:** Kruskal–Wallis analyses among 3 groups over two months of change in glycemic management status.

	Adequate to Adequate	Inadequate to Adequate	Inadequate and Adequate to Inadequate	Kruskal–Wallis Analyses among 3 Groups (*p*-Value)
Number of subjects	14	7	7	
Age, years old	70.50 [62.00, 79.25]	71.00 [64.00, 73.50]	69.00 [65.50, 79.50]	0.958
Gender (M/F)	10/4	6/1	4/3	0.497
HbA1c (0M), %	6.70 [6.45, 7.05]	7.20 [7.10, 7.65]	7.20 [7.15, 7.45]	0.022
HbA1c (2M), %	6.70 [6.32, 6.97]	6.90 [6.80, 7.05]	7.10 [7.10, 7.30]	0.020
ΔHbA1c, %	−0.15 [−0.27, −0.03]	−0.50 [−0.55, −0.30]	0.00 [−0.15, 0.20]	0.016
BMI	24.25 [23.10, 26.65]	26.20 [23.25, 28.95]	22.30 [20.75, 23.85]	0.286
Number of diabetes medications	2.00 [1.00, 2.75]	2.00 [2.00, 3.00]	2.00 [1.00, 2.50]	0.536
Subjects with diabetic complications	4	0	1	0.262
Grade of diabetes care behavior (6–18)	10.00 [7.50, 11.75]	10.00 [9.00, 12.50]	8.00 [8.00, 9.00]	0.264
Understanding of diabetes therapy (3–9)	4.00 [3.00, 4.00]	4.00 [4.00, 5.50]	4.00 [4.00, 4.50]	0.440
Acceptance of diabetes (3–6)	4.00 [4.00, 5.00]	5.00 [4.00, 5.00]	4.00 [3.50, 4.00]	0.104
Effectiveness of Robot interview (2–4)	4.00 [4.00, 4.00]	4.00 [4.00, 4.00]	4.00 [3.50, 4.00]	0.357
QC-01 (1–5)	5.00 [4.00, 5.00]	4.00 [3.50, 4.50]	5.00 [5.00, 5.00]	0.024
QC-02 (1–5)	5.00 [4.00, 5.00]	4.50 [4.00, 5.00]	5.00 [5.00, 5.00]	0.166
QC-03 (1–5)	4.50 [4.00, 5.00]	4.00 [3.00, 4.00]	5.00 [4.50, 5.00]	0.057
QC-04 (1–5)	4.00 [4.00, 4.00]	3.50 [3.00, 4.50]	5.00 [4.50, 5.00]	0.120
QC-05 (1–5)	3.50 [3.00, 4.00]	3.00 [3.00, 3.00]	4.00 [3.50, 4.50]	0.333
QC-06 (1–5)	4.00 [3.00, 4.75]	3.00 [3.00, 4.00]	4.00 [4.00, 5.00]	0.260
QC-07 (1–5)	4.00 [3.00, 4.75]	3.00 [3.00, 4.00]	5.00 [4.00, 5.00]	0.022
QC-08 (1–5)	4.00 [3.00, 5.00]	4.50 [4.00, 5.00]	5.00 [4.00, 5.00]	0.400
QC-09 (1–5)	4.50 [4.00, 5.00]	4.00 [3.50, 5.00]	5.00 [4.50, 5.00]	0.404
QC-10 (1–5)	3.00 [2.25, 4.00]	3.00 [3.00, 3.00]	3.00 [3.00, 5.00]	0.355
QC-11 (1–5)	4.00 [3.00, 4.75]	4.00 [3.00, 4.00]	4.00 [4.00, 5.00]	0.209
QC-12 (1–5)	3.00 [3.00, 3.75]	3.00 [3.00, 3.50]	3.00 [2.50, 4.00]	0.947
QC-13 (1–5)	3.00 [3.00, 3.00]	3.00 [3.00, 3.50]	3.00 [2.50, 4.00]	0.973
QC-14 (1–5)	3.00 [3.00, 3.75]	3.00 [3.00, 4.00]	3.00 [2.50, 4.00]	0.612
QC-15 (1–5)	4.00 [3.00, 4.75]	3.00 [3.00, 4.00]	4.00 [4.00, 5.00]	0.041
QC-16 (1–5)	3.50 [3.00, 4.00]	3.00 [3.00, 4.50]	5.00 [4.50, 5.00]	0.037
QC-17 (1–5)	3.00 [2.25, 3.00]	3.00 [3.00, 3.00]	3.00 [2.00, 3.00]	0.506
QC-18 (1–5)	4.00 [3.00, 4.00]	4.00 [3.50, 4.00]	4.00 [4.00, 4.50]	0.357
QC-19 (1–5)	3.50 [3.00, 4.00]	4.00 [3.00, 4.50]	4.00 [3.50, 5.00]	0.735
QC-20 (1–5)	3.00 [3.00, 4.00]	4.00 [3.50, 4.00]	5.00 [4.00, 5.00]	0.138
QC-21 (1–5)	3.00 [3.00, 4.00]	3.00 [3.00, 4.00]	4.00 [3.00, 4.50]	0.740
QC-22 (1–5)	3.50 [3.00, 4.00]	4.00 [4.00, 5.00]	4.00 [4.00, 5.00]	0.126

BMI, body mass index; M, months; QC, questionnaires categorized under Quality of Care. Clinical features as age, HbA1c, BMI, and the number of diabetes medications were expressed in mean ± S.D. The scores of diabetes self-care status, and QC01 to QC-22 were expressed as median [interquartile range].

**Table 5:** Multiple linear regression to the change in glycemic management status.

Independent Variables	Coefficient	SE	*p*-Value
HbA1c (0M), %	−0.05219	0.18283	0.7780
QC01	−0.36472	0.15504	0.0280
QC07	−0.35337	0.16189	0.0400
QC15	−0.28527	0.20757	0.1832
QC16	0.03855	0.14311	0.7901

A multivariate regression analysis was conducted with the four categories in the change in glycemic management status (GMS) (Inadequate to Adequate (I to A), Adequate to Adequate (A to A), Inadequate to Inadequate (I to I), Adequate to Inadequate (A to I)) as the dependent variable and assigned scores (I to A—4 points; A to A—3 points; I to I—2 points; A to I—1 point) along with HbA1c, QC01, QC07, QC15, and QC16 as independent variables.

## Data Availability

The datasets used and analyzed in this study are available from the corresponding author upon reasonable request.
